# Problem Gambling among Adolescent Girls in Croatia—The Role of Different Psychosocial Predictors

**DOI:** 10.3389/fpsyg.2017.00792

**Published:** 2017-05-19

**Authors:** Aleksandra Huic, Dora Dodig Hundric, Valentina Kranzelic, Neven Ricijas

**Affiliations:** ^1^Department of Psychology, Faculty of Humanities and Social Sciences, University of ZagrebZagreb, Croatia; ^2^Department of Behavioral Disorders, Faculty of Education and Rehabilitation Sciences, University of ZagrebZagreb, Croatia

**Keywords:** adolescent gambling, problem gambling, adolescent girls, risky behavior, gender specific risk factors

## Abstract

Although, compared to boys, adolescent girls gamble less often and less problematically, prevalence studies still show significant numbers of at risk/problem gamblers among girls. However, girl gambling has been on the sidelines of adolescent gambling research. The available studies usually focus only on a narrow set of correlates often ignoring that adolescent gambling is a complex phenomenon determined by various factors. Also, they often measure gambling related consequences with instruments that are not specifically developed for use on adolescents. In order to contribute to a better understanding of adolescent gambling this study focuses on problem gambling among girls. We consider different social, cognitive, motivational and behavioral factors as predictors of girl problem gambling. A total of 1,372 high-school girls from 7 Croatian cities participated in the study. They provided data on their gambling activities, peer gambling, cognitive distortions related to gambling, motivation for gambling, and levels of general risky behavior. As the only instrument developed specifically for use on adolescents, the Canadian Adolescent Gambling Inventory was used to examine adverse gambling consequences. Results show 7.4% of girls can be considered regular gamblers, and out of those who gambled at least once in their lifetime (*n* = 862), 11.2% already experience mild adverse consequences because of their gambling (at risk gamblers), with 3.2% experiencing serious consequences (problem gamblers). In general, girls seem to prefer lotto and scratch cards, but sports betting seems to be the preferred game of choice among regular girl gamblers. A hierarchical regression model confirmed the importance of much the same factors identified as risky for the development of problem gambling among adolescent boys—cognitive distortions, motives to earn money, to be better at gambling and to relax, the experiences of winning large and the drive to continue gambling, together with social factors such as having friends who also gamble, being involved in other risky and delinquent behavior and higher gambling frequency. Results call into question the importance of the motive to feel better for adolescent girls problem gambling. We discuss implications of our findings for both universal and indicated youth gambling prevention programs.

## Introduction

Studies of adolescent gambling behavior consistently show that girls are less involved in gambling than boys (Dowling et al., 2017). Also, there are less problem gamblers among girls than boys, with an estimated ratio between 1:3 and 1:5 (Jacobs, 2004). However, in spite lower prevalence rates there are several reasons why investigating gambling participation and adverse consequences in girls is important. Prevalence of both problem (1–8%) and risky gambling (1–12%) among adolescent girls is still significant (Hardoon et al., 2004; Ellenbogen et al., 2007; Donati et al., 2013; Kristiansen and Jensen, 2014). Also, girls seem to develop gambling problems more rapidly than boys, and manifest a wide array of other mental health issues in comorbidity with their gambling (Chalmers and Willoughby, 2006; Ellenbogen et al., 2007; Desai and Potenza, 2008; Jackson et al., 2008). Longitudinal data suggests that girls displaying problem gambling behavior during their adolescence remain problem gamblers in adulthood (Winters et al., 2002). Studies investigating adult gambling show consistent gender differences in gambling preferences and some gender specific pathological gambling predictors (Hing et al., 2016), leaving one to question whether these are just mirrored from female specific factors which arise in adolescence. In addition, the literature on adolescent gambling is dominated by boys, and often assumes that what is true for boy gamblers is also true for girl gamblers. At the same time, most determinants that predict adolescent problem gambling might be reliable only for boys and not girls. In line with this, several studies have questioned the utility of the same set of risk factors as predictors of girl gambling (Chalmers and Willoughby, 2006; Ellenbogen et al., 2007; Donati et al., 2013).

In order to advance the existent literature, in this study we focus exclusively on girls and examine their gambling preferences, and predictors of their problem gambling. Since problem gambling is a complex phenomenon, we consider a wide array of social, cognitive, motivational and behavioral factors, and employ a diagnostic instrument developed specifically for use on adolescents.

Girls usually start gambling in their early to middle teens, which is the same as boys (Gupta and Derevensky, 1998; Kristiansen and Jensen, 2014). Also, there seems to be no difference in overall life-time prevalence of gambling, with the majority of both girls and boys gambling at least once in their lifetime (Olason et al., 2006). Between 3 and 10% of girls seem to be regular gamblers, involving themselves in different gambling activities once a week or more often (Shapira et al., 2002; Johansson and Götestam, 2003; Skokauskas and Satkeviciute, 2007). One study even found similar numbers of adolescent boys and girls that gamble regularly (Ellenbogen et al., 2007).

Adolescent girls seem to like lotto and scratch cards as their preferred games of choice (Gupta and Derevensky, 1998; Felsher et al., 2004; Ellenbogen et al., 2007; Kristiansen and Jensen, 2014; Elton-Marshall et al., 2016). However, given the low addictive potential of lotto and scratch cards, it is possible that once they become problem gamblers girls no longer choose these games, but other more addictive gambling activities like sports betting and gaming machines. For example, studies on adults show women pathological gamblers prefer bingo and slot machines over other gambling activities (Holdsworth et al., 2012). We found only one study that examined the preferred games of choice among girl problem gamblers, which showed female problem gamblers prefer to play cards and bet on sports, together with lottery games (Ellenbogen et al., 2007). More research is needed in different geographic regions, different gambling markets and with different gambling activities before firm conclusions can be drawn.

A recent research review estimates adolescent problem gambling rates to be between 0.2 and 12.3% worldwide (Calado et al., 2016). Rates of adolescent girls' problem gambling are smaller, and differ based on the instrument used to estimate problem gambling, as well as geographic region. American and Canadian data indicates that between 1 and 3.6% satisfies the criteria for problem gambling (Derevensky and Gupta, 2000; Shapira et al., 2002; Hardoon et al., 2004; Ellenbogen et al., 2007). European data is very heterogenous, with the lowest rates of problem gambling observed among girls in Northern Europe—0.1–5.3% (Iceland—Olason et al., 2006, 2011; Denmark—Kristiansen and Jensen, 2014; Norway—Johansson and Götestam, 2003; Hanss et al., 2015; Finland—Castrén et al., 2015; Lithuania—Skokauskas and Satkeviciute, 2007). A Romanian study found much higher rates–8.3% of girls to be problem gamblers, and an additional 17.7% to be at-risk gamblers (Lupu and Todirita, 2013). In Italy, Bastiani et al. (2013) found 13.6% of girls to be moderate risk and problem gamblers. However, these prevalence rates might be misleading because they are based on a limited set of studies. Researchers interested in adolescent gambling prevalence should routinely report prevalence rates separately for boys and girls to overcome this issue.

Although these prevalence rates are smaller than rates for boys (see Calado et al., 2016) they are still relatively high, given that gambling is illegal for minors in most countries. Countries differ with regard to both accessibility and availability of gambling venues, which is one of the reasons why prevalence rates of adolescent gambling are so diverse. Given the scarcity of data on adolescent girls, further research is needed in different contexts in order to paint a full picture of girl gambling. Croatia, with its liberal gambling market, and high accessibility and availability of gambling venues, provides such a context.

Data on prevalence of gambling participation and problem gambling among adolescent girls undoubtedly indicate that girls do gamble and do so problematically and hence need both prevention and treatment interventions just as much as boys. However, studies focusing on correlates of girl problem gambling, which could inform said efforts, are very scarce and seem to be rather inconsistent. Furthermore, it is still unclear which risk factors of adolescent problem gambling are shared between boys and girls, and which, if any, are girl specific.

Most of the studies interested in gender differences examine the comorbidity of gambling and other mental health issues, although with inconsistent results. Studies focusing on gambling involvement showed that girls with more depression symptoms gamble more frequently (Gupta and Derevensky, 1998; Martins et al., 2007). However, a longitudinal study did not find depression as a significant predictor of higher gambling involvement, neither among girls, nor among boys (Yücel et al., 2015). Substance use (tobacco, alcohol, drugs) was identified as a risk factor for girls in some studies (Gupta and Derevensky, 1998; Martins et al., 2007), but not in others (Casey et al., 2011). Moreover, in a longitudinal study, only alcohol use (but not drug use) in early adolescence predicted risky gambling behavior in late adolescence, and this was true for both girls and boys (Yücel et al., 2015).

Studies that focus on problem gambling symptoms, and not just gambling frequency, also seem to be inconsistent. Desai et al. (2005) found no gender differences in alcohol and substance use, but did find higher depression rates among girl problem gamblers. In contrast, Ellenbogen et al. (2007) found no gender differences in rates of depression and alcohol use, but did find higher rates of drug use among girl problem gamblers.

According to some authors, gambling can be considered as part of the general problem behavior framework (Dickson et al., 2002; Barnes et al., 2011) and numerous studies confirm that gambling behavior occurs in comorbidity not just with substance use, but with general risky and anti-social behavior as well (Wanner et al., 2009; Dowling et al., 2017; Mishra et al., 2017). However, studies comparing boys and girls on this issue are very scarce, and include only measures of gambling frequency and not problem gambling. For example, Jackson et al. (2008) found anti-social behavior to be a risk factor for higher gambling involvement among boys, but not girls. On the other hand, in a longitudinal study, peer delinquency predicted gambling participation only among girls, and not boys (Barnes et al., 2005). Another longitudinal study, although focused only on adolescent boys, also showed that levels of aggressive and disruptive behavior in childhood and early adolescence predicted at risk/problem gambling in later adolescence (Martins et al., 2013). However, further research that focuses on problem gambling and not just gambling frequency is needed before any conclusions on the role of risky and delinquent behavior for girl gambling/problem gambling can be drawn.

Desai et al. (2005) speculate that girl gambling reflects a particularly deviant path characterized by a complicated clinical picture involving disrupted mental health, especially mood disorders and substance abuse. Going into adulthood, gambling in order to overcome mental health problems might be the primary reason for women gambling (Thomas and Moore, 2001). Several studies show that women are more likely to use gambling as an escape from worry and other life problems, while men seem to gamble in order to win money and because they believe that they can influence the outcome (Wenzel and Dahl, 2009; Balodis et al., 2014). In contrast to gender differences in adult gambling motivation, studies on adolescents seem to show different patterns. Boys endorse most of the reasons for gambling (to make money, escape problems, to feel better, to be entertained) more than girls (Jackson et al., 2008; Dodig, 2013; Kristiansen and Jensen, 2014).

However, if research wants to inform future prevention and treatment efforts, an important question is whether different motives predict problematic gambling in different ways for boys and girls. With this regard studies on adult gamblers show much less gender differences. Several studies found that expectations to win money and to have a good time predicted gambling problems for both women and men (Spurrier and Blaszczynski, 2014; Hing et al., 2016). Gambling to escape problems seems to be the only consistent motive that predicts women's but not men's gambling problems (Walker et al., 2005). However, we were not able to find studies that investigate gender differences regarding the contribution of motivation for problem gambling in adolescence, so future research is needed on this point.

It is clear from this short review of available research on gender differences in adolescent gambling, that our knowledge of female gambling, especially female problem gambling is very limited. In addition, current findings seem to be rather inconsistent, and further research is needed before any of the factors associated with adolescent problem gambling can be branded as girl specific.

In this study we try to extend the available literature by focusing solely on girls. We had two main goals—to examine gambling participation and preferences among Croatian adolescent girls and girl problem gamblers, and to investigate whether risk factors commonly associated with adolescent problem gambling predict girl problem gambling as well. In order to do so, we employed a comprehensive model of cognitive, motivational, social and behavioral factors which we based on previous research showing the most common predictors of adolescent problem gambling (Derevensky and Gilbeau, 2015), and other Croatian studies investigating adolescent problem gambling (Dodig, 2013; Ricijas et al., 2016a).

We included different types of cognitive distortions as *cognitive factors* in our model. Previous studies on adolescents show that distortions such as poor understanding of odds and probabilities, superstitious thinking and illusions of control predict more adverse gambling consequences and problems (Goodie and Fortune, 2013), and this was found for both boys and girls (Donati et al., 2013). In line with these studies, we expected different types of cognitive distortions to significantly predict girl problem gambling.

As *motivational factors*, we were interested in motives to win money, to be better at gambling, to feel better and to relax. These motives have been linked to more adverse gambling consequences and problems in previous research (Derevensky and Gilbeau, 2015). Even though research on adults shows women primarly gamble to feel better, and not to earn money, based on the available studies with adolescents where much less gender differences were observed, we expected that all four motives will predict more severe gambling problems in girls. Furthermore, previous studies showed that experiencing large wins leads to a specific motivation or drive to continue gambling after winning and that this specific motivation is one of the strongest single predictors of gambling problems (Turner et al., 2006; Ricijas et al., 2016a). In line with this, we expected that both previous large wins experience and the drive to continue gambling after winning to be potent predictors of girl problem gambling.

We also included *social factors*, namely peer gambling involvement, in our study. Although peer gambling has been associated with adolescent problem gambling in previous research (Delfabbro and Thrupp, 2003; Hardoon et al., 2004; Langhinrichsen-Rohling et al., 2004; Dickson et al., 2008), studies investigating girl specific problem gambling predictors generally overlook these factors. One study (Donati et al., 2013) found peer gambling to predict problem gambling only among boys, but not girls. On the other hand, some authors argue that peer related factors might be more important for girls than for boys (Chalmers and Willoughby, 2006). However, peer influence is a strong factor that shapes adolescent behavior (Ryan, 2001), especially risky behavior and risky decision making (Chassin et al., 2004; Gardner and Steinberg, 2005; Simons-Morton et al., 2005). Since gambling is often considered part of the general problem-behavior framework (Dickson et al., 2002; Barnes et al., 2011), we expected that having friends that gamble will be tied to more problematic girl gambling.

We examined gambling frequency and other risky behaviors, as *behavioral factors*. In general, strong links exist between levels of risky and delinquent behavior and adolescent problem gambling (Wanner et al., 2009; Welte et al., 2009), although we were not able to find studies examining gender differences in this link. Studies on delinquent girls who are involved in risky behavior during their adolescence show high comorbidity between a wide array of different risky and antisocial behaviors (Zahn et al., 2010). Based on this, we expected that levels of general risky and delinquent behaviors will be associated with more problematic gambling among adolescent girls.

Gambling frequency is one of the strongest predictors of problem gambling (Boldero et al., 2010; Raisamo et al., 2013). Although frequency alone is not enough to diagnose problematic gambling, adolescents who gamble more frequently routinely show more adverse gambling consequences and problems, so we expected the same will be true for adolescent girls. Similarly, those who exhibit more gambling problems also seem to be involved in a wider array of gambling activities (Kristiansen and Jensen, 2014), something we also expected to find on girls.

All the available studies examine girl gambling or gender differences in gambling by relying on commonly used screening instruments like DSM-IV-MR-J (Fisher, 2000) or SOGS-RA (Winters et al., 1993). Since these have been adapted from conceptualizations of adult gambling, they have been criticized for not adequately capturing adolescent problem gambling (Stinchfield, 2010; Dodig, 2013). In order to investigate different kinds of gambling related consequences and harms, typically reported by adolescents, in this study we use the only existing screening instrument developed for use especially on adolescents. The Canadian Adolescent Gambling Inventory (CAGI; Tremblay et al., 2010) recognizes that adolescents can have different psychological, social, behavioral and financial costs because of their gambling, and takes all of these into account when categorizing no-risk, at-risk and problem gamblers. Using this instrument to investigate girl problem gambling enables us to further extend the available literature.

## Method

### Participants

A total of *N* = 1,372 high-school girls from 7 Croatian cities participated in the study. Both major Croatian regional centers, as well as smaller Croatian towns were included. In regional centers (Zagreb, Rijeka, Osijek, Split), high-schools were randomly selected from the list of available schools in the city. In smaller towns, a convenient sample of schools was used. Age ranged from 14 to 20 years (*M* = 16.45, *SD* = 1.154). All three Croatian high-school programs (general education, 3-year and 4-year vocational schools), and all levels of high-school grades are represented in the sample. In terms of high school programs, each category closely represents the national distribution of the number of students enrolled in these programs. Also, there is an approximately equal number of first, second and third grade students while, due to the absence of the fourth grade in 3-year high-school programs, the proportion of students who attend the fourth grade is slightly lower. The basic socio-demographic characteristics of the sample are presented in Table 1.

Table 1**Sample description (***n*** = 1,372 high-school girls)**.

**Table:** 

**City/Town**	**Zagreb**	**Split**	**Rijeka**	**Osijek**	**Koprivnica**	**Slavonski brod**	**Vinkovci**
***N***	282	321	210	220	109	117	113
**%**	20.6	23.4	15.3	16.0	7.9	8.5	8.2

**Table:** 

**Type of school**	**3-year vocational school**	**4-year vocational school**	**General education**
***N***	247	452	673
**%**	18.0	32.9	49.1

**Table:** 

**Grade**	**1st**	**2nd**	**3rd**	**4th**
***N***	411	353	335	268
**%**	30.1	25.8	24.5	19.6

### Measures

**General socio-demographic information** such as age, grade and gender were collected.

We used the revised version of **Gambling Activities Scale** (Ricijas et al., 2011) to assess gambling frequency. We focused on six games: (1) sports betting, (2) lotto, (3) scratch cards, (4) slot machines, (5) electronic roulette, and (6) betting on virtual races. The respondents were asked to evaluate the frequency of their gambling (on a 5 point scale, from *0* = *never* to *5* = *every day or almost every day*). In order to assess the overall frequency of their gambling, we averaged the result for all games of chance. Higher results indicate more frequent gambling with the reliability of this index being satisfactory (α = 0.62).

The severity of gambling related consequences was measured by the **Canadian Adolescent Gambling Inventory (CAGI)** (Tremblay et al., 2010). It is the first instrument designed specifically for the assessment of adolescent problem gambling. Therefore, its items are in line with the developmental age of the respondents (item example: “How often have you taken money that you were supposed to spend on lunch, clothing, movies, etc. and used it to gamble/bet or to pay off your gambling/betting debts?”), who report how often they felt or behaved in a certain way on a 4 point scale (*0* = *never* to *3* = *almost always or 7 and more times*). In this study we used only the nine items which provide a composite measure, a General Problem Severity Subscale (GPSS) (α = 0.79). Depending on the total result of this measure, participants are classified into 3 categories: (1) no problem (“green light”), (2) low to moderate severity (“yellow light”), and (3) high severity (“red light”). Several studies confirm good classification accuracy of the GPSS measure–Se = 0.97 and Sp = 0.93 (Tremblay et al., 2010); Se = 0.93, Sp = 0.99 and hit rate = 0.98 (Jiménez-Murcia et al., 2017).

The **Gambling Beliefs Scale** (Ricijas et al., 2011) is a 14-item scale which measures gambling related cognitive distortions. There are two subscales: (1) Superstition and incorrect understanding of chances and probability (9 items, α = 0.81 item example: “*Some activities (rituals etc.) increase the probability of winning at gambling.”*) and (2) Illusion of control (5 items, α = 0.74, item example: “*Gambling outcomes can be predicted.”*). The respondents report on their level of agreement with each of the items on a four-point scale ranging from *1 (strongly disagree)* to *5 (strongly agree)*. The scale provides two separate results, one for each factor with higher results indicating more cognitive distortions associated with gambling.

**Risk and delinquent behavior** was measured with a self-report scale by Atlanta et al. (2005). In this 25-item scale, various types of risk and delinquent behavior are listed (for example stealing, vandalism, aggressive behavior etc.) and the respondents indicate how many times in their life have they done something or behaved in a certain way (*0* = *never, 1* = *one to two times, 2* = *three to four times, 3* = *five or more times*). The item scores are averaged to form a total score. Again, a higher result indicates more involvement in such behavior. Cronbach alpha was α = 0.87.

**Motives for Gambling Check-List** (Ricijas et al., 2011) was created to assess motivation for gambling. In this study, the participants were asked “Why do you gamble/bet?,” and then offered four potential motives (to relax, to feel better, to earn money, to become better in gambling). For each of them, participants indicated how often they gamble because of that specific motive *(0* = *never because of that; 4* = *always because of that)*. The emphasis is on these 4 motives only since previous studies have shown them to be especially significant when it comes to explaining gambling related problems (Gupta and Derevensky, 1998; Moore and Ohtsuka, 1999; Delfabbro and Thrupp, 2003; Wood and Griffiths, 2007; Yip et al., 2011; Derevensky and Gilbeau, 2015).

Furthermore, respondents were asked about their **peers' gambling** habits. Specifically, they were asked to remember a few of their closest friends and indicate how often (*0* = *never to 5* = *every day)* they think their friends are involved in (a) sports betting, (b) slot machines, and (c) electronic roulette. A total score was formed by summing responses on all three items creating a peer gambling index. Higher results indicate higher instances of peer gambling. The reliability of such an index was satisfactory α = 0.76.

Since **experiences while gambling** are particularly relevant for the development and maintenance of problem gambling, we wanted to gain insight into participants' feelings of experiencing reinforcement while gambling. Therefore, we asked them: (1) “How often have you had an experience of winning a large sum of money while gambling?” *(1* = *never; 4* = *many times)* and (2) “When winning a large sum of money by gambling it encourages me to continue gambling.” *(1* = *not at all; 5* = *completely true for me)*.

### Procedure

The data was collected in school classrooms, during regularly scheduled classes. Consent to participate was considered implicit by the return of the questionnaire, after the participants were fully informed about the study. Students could decline participation at any point during instrument administration. The researcher distributed the paper-pencil surveys and students completed them independently and anonymously. The full survey took ~45 min to complete. During all of the procedures, the principles of the Code of Ethics for Research with Children (Ajdukovic and Kolesaric, 2003) were respected. The ethics committee of the Faculty of Education and Rehabilitation Sciences, University of Zagreb approved the research. Furthermore, the study had written support from Croatian Ministry of Education, Science and Sport and Croatian Teacher Training Agency. Afterwards, all participating schools received a descriptive summary report of results on a group level.

### Statistical analyses

Data was analyzed using the statistical package SPSS version 20.0 (SPSS Inc., Chicago, IL). We calculated descriptive statistical parameters in order to analyze frequency of gambling and problem gambling. One-way ANOVA was used to examine differences in gambling frequency among different categories of gamblers. We examined associations between study variables with Pearson's correlation coefficients. We employed hierarchical regression analyses in order to test which correlates significantly predicted problem gambling among Croatian adolescent girls.

## Results

### Descriptives

First, we examined the frequency of engaging in the six gambling activities (sports betting, lotto, scratch cards, slot machines, electronic roulette, and virtual betting). Based on criteria proposed by Felsher et al. (2004) we categorized girls gambling activities in three categories—never played; played occasionally (combining answers “once a year or less” and “once a month”); and played regularly (once a week or more often). A total of 7.4% can be considered regular gamblers, playing at least one of the six examined activities once a week or more often. Data on specific activities, and preferred games of choice for Croatian high-school girls can be seen in Table 2.

**Table 2:** **Frequency of Gambling (***N*** = 1,372)**.

	**Never**		**Occasionally**		**Regularly**	
	***f***	**%**	***f***	**%**	***f***	**%**
Sports Betting	1,145	83.5	189	13.8	38	2.8
Lotto	886	64.6	457	33.3	29	2.1
Scratch Cards	701	51.1	638	46.5	33	2.4
Slot machines	1,199	87.4	152	11.1	21	1.5
Electronic roulette	1,344	98	23	1.7	5	0.4
Betting on virtual races	1,296	94.5	62	4.5	14	1.0

Scratch cards and lotto are the most common games played by girls, with significant numbers of girls betting on sports and playing slot machines. A third of girls occasionally play lotto, and about half of them occasionally play scratch cards. An additional 2% of girls play these two games regularly. Over 10% of girls bet on sports occasionally, with almost 3% betting on sports regularly. It is interesting that amongst the games that are played regularly, sports betting is at the top of the hierarchy, together with lotto and scratch cards. Slot machines are also fairly common, with 11% of girls playing this game occasionally and an additional 1.5% regularly. Betting on virtual races and electronic roulette seem not to be preferred by girls.

According to the CAGI general problem gambling severity index (GPSS), the majority of girls are not experiencing any adverse gambling related consequences (91.6%). However, 1.7% of girls are already experiencing serious consequences because of their gambling (“red light”), and 6.7% are experiencing low to moderate consequences (“yellow light”). This means almost 10% of high-school girls are at risk to develop or have already developed gambling problems (see more on boy and girl gambling prevalence in Ricijas et al., 2016b).

However, when we look only at those girls who have gambled at least once in their lifetime (*N* = 862) the numbers are more alarming—11.2% are experiencing low to moderate consequences and 3.2% are already experiencing severe consequences because of their gambling. In Table 3 we present information on preferred games of choice based on different CAGI categories. Only games played regularly (once a week or more often) are included. Results show that amongst girls with severe gambling related consequences slot machines and sports betting seem to be the preferred games of choice, with a third of girls being involved in these activities. Scratch cards, being one of the most frequently played game overall (see Table 2), is hardly represented among those girls who already have severe gambling related consequences, with only one seventh of at risk gamblers (yellow light) regularly playing this game.

**Table 3:** **Frequency of regular gambling according to CAGI categories (***N*** = 862)**.

	**CAGI green light**		**CAGI yellow light**		**CAGI red light**	
	***f***	**%**	***f***	**%**	***f***	**%**
Sports Betting	15	2.1	16	16.8	7	25.9
Lotto	15	2.1	11	11.6	3	11.1
Scratch Cards	20	2.8	12	12.6	1	3.7
Slot machines	11	1.5	2	2.1	8	29.6
Electronic roulette	1	0.1	2	2.1	2	7.4
Betting on virtual races	7	1.0	4	4.2	3	11.1

We also examined differences between CAGI categories in the number of different games girls play regularly. Results show that social gamblers (green light) have one preferred game of choice, with only 1.5% choosing to play two or three games regularly. Among at-risk gamblers (yellow light) there is 12.7% who play between two and four different games regularly. And among problematic gamblers (red light) 18.5% play between two and four different games regularly. In this sample none of the girls play more than four of the examined games regularly. We calculated a one-way ANOVA using the number of games they play regularly. The analysis showed a significant difference between CAGI categories (*F* = 60.886, *p* < 0.0001). *Post-hoc* testing showed problematic gamblers playing the most games regularly, followed by at-risk gamblers and then non-risk gamblers (*p's* between all groups < 0.001).

### Predictors of adverse gambling consequences among girls

Our second goal was to examine different cognitive, motivational, social, and behavioral predictors of problem gambling among adolescent girls. Descriptives and correlations between all predictors and problem gambling are presented in Table 4. We did this, and all subsequent analyses, only on the subsample of girls who gambled at least once in their lifetime, since one needs to be gambling in order to experience any adverse gambling related consequences.

**Table 4:** **Descriptives and correlations between study variables (***N*** = 862; high-school girls who gambled at least once in their life-time)**.

	**CAGI**	**RISK**	**PG**	**COGDIS1**	**COGDIS2**	**M1**	**M2**	**M3**	**M4**	**Experience 1**	**Experience 2**	**FG**
CAGI	−	0.336^**^	0.249^**^	0.202^**^	0.185^**^	0.413^**^	0.379^**^	0.361^**^	0.464^**^	0.470^**^	0.401^**^	0.418^**^
Risk and delinquent behavior (Risk)		−	0.255^**^	0.165^**^	0.149^**^	0.226^**^	0.255^**^	0.313^**^	0.357^**^	0.225^**^	0.229^**^	0.287^**^
Peer gambling (PG)			−	0.084^*^	0.039	0.130^**^	0.142^**^	0.139^**^	0.171^*^	0.218^**^	0.183^**^	0.248^**^
Superstition and incorrect understanding of chances and probability (COGDIS1)				−	0.567^**^	0.277^**^	0.288^**^	0.149^**^	0.314^**^	0.203^**^	0.186^**^	0.140^**^
Illusion of control (COGDIS2)					−	0.194^**^	0.178^**^	0.152^**^	0.261^**^	0.159^**^	0.174^**^	0.119^**^
Motive to relax (M1)						−	0.530^**^	0.204^**^	0.464^**^	0.319^**^	0.294^**^	0.298^**^
Motive to feel better (M2)							−	0.332^**^	0.514^**^	0.315^**^	0.320^**^	0.268^**^
Motive to earn money (M3)								−	0.269^**^	0.349^**^	0.434^**^	0.359^**^
Motive to be better in gambling (M4)									−	0.319^**^	0.345^**^	0.336^**^
Experience of winning a large sum of money (Experience 1)										−	0.282^**^	0.500^**^
Drive to continue with gambling after winning a large sum of money (Experience 2)											−	0.277^**^
Mean frequency of gambling (FG)												−
Mean	0.75	1.69	4.65	1.86	2.60	2.21	1.18	2.04	1.19	1.38	1.91	2.69
SD	1.972	0.398	4.070	0.639	0.784	0.570	0.504	1.120	0.596	0.728	1.183	2.289
Minimum	0	1	0	0	1	1	1	1	1	1	1	1
Maximum	17	3.75	15	4.6	5	4	4	4	4	4	5	19
Theor. range	0–27	1–4	0–15	1–5	1–5	1–4	1–4	1–4	1–4	1–4	1–5	0-30

* p < 0.050;

** *p < 0.010*.

Bivariate correlations between predictors and gambling related consequences are all significant and in expected directions. Those with more cognitive distortions, higher motivation and drive for gambling, who gamble more and have friends who gamble more, and those with higher levels of risk and delinquent behavior have more adverse consequences because of their gambling. The predictors themselves are also intercorrelated, so further analysis is warranted in order to control for this shared variability.

We grouped our predictors based on the content of what they are measuring—cognitive, motivational, social, and behavioral factors. Then we decided to enter them into a six-step hierarchical regression analysis, entering variables most distant from gambling first (risk and delinquent behavior in step 1; peer gambling in step 2), moving to the factors more proximal to gambling (cognitive distortions related to gambling in step 3; motives for gambling in step 4; experiences while gambling in step 5, and gambling frequency in step 6). This strategy takes into account the complexity of problem gambling, and allows us to examine the different predictive value of specific problem gambling determinants.

Results are presented in Table 5. The entire model explains 41.5% of adverse gambling consequences among adolescent girls. All VIFs ranged from 1.079 to 1.661 showing no multi-collinearity problems in the analysis. Every step significantly improved prediction when entered into the analysis. Also, all predictors, except for cognitive distortions, remained significant in the last step. Girls with higher levels of risk and delinquent behavior suffer from more adverse gambling consequences, and so do girls who surround themselves with close friends who gamble often. Girls with more gambling related cognitive distortions such as incorrect understanding of probability and chance, more superstitious beliefs, and higher illusion of control all exhibit more adverse gambling consequences. However, specific motives for gambling were the most potent individual predictors of adverse consequences (explaining almost 20% of the entire variance by themselves). Thus, when we entered them into the analysis, cognitive distortions lost their predictive strength. Out of the four motives, specific motivation to earn money by gambling, and to become better at gambling, as well as to gamble in order to relax, proved to be significant individual predictors. Motivation to feel better was not a significant predictor of adverse gambling consequences. As expected, those who had more experience winning large sums of money (according to their own subjective feeling of what a large sum of money is) and those with the drive to continue gambling while winning also experience more adverse gambling consequences, and so do girls who gamble more often.

**Table 5:** **Results of hierarchical regression analysis**.

		**Step 1**	**Step 2**	**Step 3**	**Step 4**	**Step 5**	**Step 6**
		**β**	**β**	**β**	**β**	**β**	**β**
1	Risk and delinquent behavior	0.340^***^	0.291^***^	0.263^***^	0.111^***^	0.093^**^	0.078^*^
2	Peer gambling		0.181^**^	0.179^**^	0.130^**^	0.088^**^	0.075^*^
3	Superstition and incorrect understanding of probability and chance			0.091^*^	−0.022	−0.031	−0.027
	Illusion of control			0.101^*^	0.056	0.041	0.041
4	Motive to earn money				0.183^***^	0.086^**^	0.065^*^
	Motive to relax				0.204^***^	0.155^***^	0.141^***^
	Motive to be better in gambling				0.233^***^	0.190^***^	0.176^***^
	Motive to feel better				0.049	0.025	0.029
5	Experience of winning a large sum of money					0.227^***^	0.180^***^
	Drive to continue with gambling after winning a large sum of money					0.147^***^	0.144^***^
6	Mean frequency of gambling						0.134^***^
	Total model						
	*R*	0.340	0.382	0.417	0.595	0.642	0.651
	*Adj. R^2^*	0.114	0.144	0.170	0.347	0.404	0.415
	*ΔR^2^*	0.116^***^	0.030^***^	0.028^***^	0.180^***^	0.059^***^	0.011^***^

β, standardized beta coefficient; R, multiple correlation coefficient; Adj. R^2^, the adjusted coefficient of determination; ΔR^2^, R^2^ change;

* p < 0.050;

** p < 0.010;

*** *p < 0.001*.

## Discussion

Findings on gambling participation of Croatian adolescent girls show that, just like in other countries, girls in Croatia are involved in gambling activities, and part of them develop gambling related problems. However, it seems that girls have somewhat different preferences compared to boys. Most girls in our sample play lotto and scratch cards. This confirms findings from other studies showing that lotto and scratch cards seem to be the preferred games of choice among adolescent girls (Gupta and Derevensky, 1998; Volberg, 1998; Jacobs, 2000; Stinchfield, 2000; Felsher et al., 2004; Ellenbogen et al., 2007; Kristiansen and Jensen, 2014; Elton-Marshall et al., 2016). In contrast, adolescent boys prefer sports betting, slot machines and virtual betting, both in Croatia (Ricijas et al., 2016b) and in other countries (Felsher et al., 2004; Ellenbogen et al., 2007; Kristiansen and Jensen, 2014; Elton-Marshall et al., 2016). This difference in gaming preferences helps explain stark differences in the prevalence of girl and boy problem gambling. Slot machines and sports betting are games characterized by high event frequency, “near misses” and perceived elements of knowledge/skills and have been found to be especially risky and associated with problem gambling (Griffiths, 2000; Reith, 2006). With this regard lotto and scratch cards seem to have a lower addictive potential, although their potential adverse consequences should not be ignored. They provide a window into gambling life for adolescent girls, and can spark their curiosity for other gambling activities. At the same time people tend not to perceive them as potentially harmful and do not believe they can lead to problems, which is a myth (Ariyabuddhiphongs, 2011). In reality, there are cases of scratch-cards gambling disorders (Raposo-Lima et al., 2015). Moreover, some authors argue that scratch cards, with their potential high event frequency, also have addictive potential, and go so far as to calling them “paper slot machines” (Griffiths, 2005). Given these findings it would be wrong to conclude that girls playing lotto and scratch cards are not at any risk to develop gambling problems.

Percentages of girls who gamble regularly (once a week or more often) are rather low, especially when compared to their male counterparts in Croatia (Ricijas et al., 2016b). However, a bit over 7% of girls can be viewed as regular gamblers, which is a number roughly comparable to other countries (Shapira et al., 2002; Johansson and Götestam, 2003; Olason et al., 2006; Skokauskas and Satkeviciute, 2007). These numbers are not surprising given the Croatian liberal gambling market. In spite of gambling being illegal for minors (adolescents younger than 18) a high number of gambling venues in Croatia remain both available and accessible to teens (Ricijas et al., 2016b).

Moreover, 3% of girls regularly bet on sports, and among the games that are played regularly sports betting is just as common as lotto and scratch cards. It is interesting how, compared to adult women who seem to prefer slot machines over other gambling activities (Hing and Breen, 2001; Potenza et al., 2001; Holdsworth et al., 2012), this type of game is not widespread among Croatian adolescent girls. Although when both regular and occasional gambling on slot machines is taken into account, numbers of girl gamblers who like this activity is again rather high, especially when illegality of machines is considered.

Prevalence rates in our sample confirm that numbers of at-risk and problem gamblers among girls are substantial. Among the entire sample, almost 2% of girls are already experiencing serious consequences because of their gambling (can be considered problem gamblers), and almost 7% are experiencing low to moderate consequences (at-risk gamblers). This data can be used as an indicator of general prevalence, however when we look only at those girls who have gambling experience, the numbers of at-risk (11.2%) and problem gamblers (3.2%) are more alarming and point to the necessity of prevention and treatment interventions efforts to be aimed at girls, just as well as at boys. Our data are roughly comparable to other studies (Hardoon et al., 2004; Ellenbogen et al., 2007; Donati et al., 2013; Lupu and Todirita, 2013; Kristiansen and Jensen, 2014), although one has to be very careful in this particular comparison because we used a different screening instrument to identify at-risk and problem gamblers.

Furthermore, girls with more severe gambling related consequences no longer prefer just one type of gambling activity, but are prone to play several games regularly (once a week or more often). In general, adolescent problem gamblers participate in more different gambling activities than non-risk gamblers (Kristiansen and Jensen, 2014), and our data shows adolescent girls also follow this trend.

Most interestingly, although lotto and scratch cards seem to be the preferred games of choice for adolescent girls overall, our findings are completely different when we look at preferences of girl problem gamblers. Here we see slot machines and sports betting to actually be the preferred games of choice. These findings are expected when the addictive potential of these types of activities is taken into account. Also, studies show that games most commonly played by adolescent problem gamblers are slot machines, sports betting and card games (see Calado et al., 2016 for review). With the exception of card games, which is not a common gambling activity in Croatia, neither for boys or girls (Dodig and Ricijas, 2011), Croatian girl problem gamblers seem to prefer the same games as boy problem gamblers.

Our second goal was to investigate the predictive power of different cognitive, motivational, social and behavioral factors for adolescent girls' problem gambling. Our results are mainly in line with other research investigating general problem gambling correlates among adolescents (Derevensky and Gilbeau, 2015) and confirm the importance of all these factors for girls. Girls who display higher levels of general risky and delinquent behavior also have more severe consequences because of their gambling. Our measure of risky behavior was rather comprehensive and included different behaviors such as stealing, vandalism, aggressive behavior, truancy, substance use etc. However, future studies might examine the importance of specific risky and delinquent behavior. If girl problem gamblers are to be differentiated between boy problem gamblers, it would probably be at the level of specific behaviors. For example, boys not only commit more delinquent and criminal acts, but are typically involved in more serious offenses (Zahn et al., 2010). It is possible that problem gambling among boys is tied to more serious delinquent behavior, while problem gambling among girls is more tied to risky behavior, especially in the school setting, as some authors seem to indicate (Casey et al., 2011).

Our finding that girls whose friends also participate in gambling activities already exhibit more severe gambling consequences and problems, is in line with research on boys (Ricijas et al., 2016a), with other studies of adolescent problem gambling (Delfabbro and Thrupp, 2003; Hardoon et al., 2004; Langhinrichsen-Rohling et al., 2004; Dickson et al., 2008), and with research showing that having friends who are also involved in risky and problem behavior increases the chances of the adolescent to also become problematic (Chassin et al., 2004; Gardner and Steinberg, 2005; Simons-Morton et al., 2005). However, our results are not in-line with Donati et al. (2013) study in which peer gambling involvement was not a significant predictor of girl problem gambling. Explanation of these different results probably lies in the different measures of peer involvement, and future studies should adopt similar, reliable and valid measures before firm conclusions about the role of peer involvement for girl problem gambling. Future studies might also want examine peer gambling more closely, specifically investigate characteristics of peer gamblers—are they also problem gamblers, or just occasional gamblers, what is their gender and are there gender differences in having same-sex vs. opposite sex peer gamblers. Moreover, gambling-related perceived norms have been linked to higher gambling involvement in previous studies (Foster et al., 2014; St-Pierre et al., 2015). However, we do not know if perceived norms and peer gambling influence the development of gambling related problems in the same way for girls and boys.

As expected, both cognitive and motivational factors play important roles in predicting girl problem gambling. When first added to the model, both types of cognitive distortions were significant. Girls with higher illusions of control, and those with poorer understanding of probabilities and higher levels of superstitious thinking, display more severe gambling consequences. This is in line with other research on adolescents in general (Derevensky and Gilbeau, 2015), and in girls specifically (Donati et al., 2013). However, when motivational factors were added to the model cognitive factors were no longer significant. This is in accordance with numerous studies showing that cognitive and motivational factors are inextricably linked (Moore and Ohtsuka, 1999; Delfabbro et al., 2006; Marmurek et al., 2014), and that cognitive factors depend strongly on motivational factors (Thompson et al., 2007). A Croatian study which involved only adolescent boys showed similar results (Ricijas et al., 2016a).

Just like in the Ricijas et al. (2016a) study, findings on girls similarly show that motivational factors are the strongest predictors of problem gambling. Girls with a higher motivation to earn money, to be better at gambling and to relax, just like girls with more experiences of winning large sums of money and the drive to continue gambling after winning have more severe gambling consequences. On the other hand, the motive to feel better did not significantly predict girl problem gambling. To the best of our knowledge, ours is the first study to investigate different motives as predictors of girl problem gambling, and our results are mainly in line with studies showing these particular sets of motives to be especially salient among problem gamblers (Wood et al., 2004; Yip et al., 2011; Spurrier and Blaszczynski, 2014; Derevensky and Gilbeau, 2015; Hing et al., 2016).

However, they are in contrast with research on adults which shows that women gamble to alleviate their stress, loneliness and depression (Wenzel and Dahl, 2009), in other words in order to feel better. A number of studies on adult women and their motives was done on treatment samples, and it is reasonable to assume that our study involved mainly healthy and no-problem adolescent girls (as witnessed by levels of problem gambling and risky and delinquent behavior). Future studies might want to investigate gambling motivation among girls already being treated for gambling problems. The motive to feel better might be predominant among this specific population.

As expected from other studies, higher gambling frequency predicted more severe gambling consequences in girls, just like it predicts the same adverse consequences in boys (Ricijas et al., 2016a). Logically, those who gamble more frequently have more opportunities to experience both positive and negative outcomes of gambling involvement, which can then be associated with different psychological consequences and loss of control, disrupted peer and family relationships, as well as different financial consequences.

Each set of factors, when first introduced into our model, significantly improved prediction and added to the overall variance explained. This confirms that adverse gambling consequences depend on a complex set of factors, just like many other studies of gambling emphasize (Blaszczynski and Nower, 2002; Nower and Blaszczynski, 2004). On the other hand, considering the high number of predictors, the overall percentage of variance explained is rather low (just over 40%). Similar sets of factors in other studies, dominated by boys, explain larger percentages of variance (Clarke, 2004; Ricijas et al., 2016a). This probably means that although our study shows that girl problem gambling is predicted by largely the same factors as boy problem gambling, there might be other determinants not investigated here that are possibly girl specific and better capture adolescent girl gambling consequences. Therefore, and based on the findings of previous research, it would be interesting to explore the contribution of mental health issues (Potenza et al., 2001; Desai et al., 2005), childhood trauma (Petry and Steinberg, 2005), coping mechanisms (Afifi et al., 2010) and family influences (such as family functioning, mental health problems of family members, their gambling etc.; Hardoon et al., 2004; Langhinrichsen-Rohling et al., 2004; Chalmers and Willoughby, 2006; McComb and Sabiston, 2010).

Our study has several limitations that need to be taken into account. Firstly, our design was cross-sectional and we rely on self-reports, both of which limit our conclusions. Because of this, it is equally likely that all the examined factors lead to problem gambling, as it is likely that problematic gambling involvement leads to certain cognitions, motives, and behavior. Only future longitudinal studies can settle this issue. Our results are nevertheless important because they show the need to investigate girl problem gambling in more depth.

Moreover, our screening instrument was developed on both girls and boys, but there is no available data on its gender invariance. Since some other studies indicate that boys and girls might understand items in instruments differently, and question their validity for girls (Derevensky and Gupta, 2006; Rossow and Molde, 2006) this is another potential limitation of our study. However, we did use an instrument developed specifically for use on adolescents, which captures consequences linked to girl problem gambling in previous studies (Wiebe et al., 2000; Ellenbogen et al., 2007), which we believe to be one of the strengths of our study. Future research should test for gender invariance of the CAGI, and other commonly used screening instruments, in order to be able to reliably test for gender differences in adolescent problem gambling.

## Conclusion

Although gambling participation and prevalence of problem gambling is more widespread among boys than girls worldwide (Calado et al., 2016), results of this study show that girls still gamble and develop gambling related consequences and problems. Our findings indicate that prevention efforts need to be aimed at girls just as much as at boys. One widely discussed question in planning prevention interventions is whether to employ any gender specific approach/strategy or focus on both boys and girls in the same manner (Blake et al., 2001; Rohrbach and Milam, 2006; Vigna-Taglianti et al., 2009). Our results indicate that universal youth gambling prevention programs (ones focused on general populations) might want to employ some gender specific strategies. Given the differences between the preferred games of choice among girls and boys (lotto and scratch cards vs. sports betting), universal programs should teach about perils of all these gambling activities if they want to be effective for both girls and boys.

However, our results also indicate that girls with gambling problems prefer games like slot machines and sports betting, which is a point of similarity between girls and boys. Our study also shows adverse gambling consequences experienced by girls are predicted by a set of complex factors which include cognitive distortions, motives to earn money, be better at gambling and to relax, the experiences of winning large and the drive to continue gambling, together with social factors such as having friends who also gamble, being involved in other risky and delinquent behavior and higher gambling frequency. All these have been identified as risk factors of boys gambling in previous research (Derevensky and Gilbeau, 2015), and our findings confirm the relevance of the same risk factors for girl problem gambling.

Consequently, our findings show that youth gambling prevention programs at the indicated level (ones designed for populations at high risk and/or populations already exhibiting gambling related problems) should refrain from tailoring activities to be boy or girl specific. However, studies focusing on girl gamblers and girl specific predictors are still very scarce, and much future research is needed before firm conclusions on these issues can be drawn. For example, if girls really tend to exhibit a particularly complicated clinical picture characterized by high comorbidity with other mental health issues (Desai et al., 2005), indicated prevention programs than need to take this gender specificity into account. More longitudinal studies are needed in order to reach reliable conclusions on any gender specific risk and protective factors of adolescent problem gambling.

## Ethics statement

The study was carried out in accordance with the latest version of the Declaration of Helsinki and the Croatian Code of Ethics for Research with Children (Ajdukovic and Kolesaric, 2003). The Ethics Committee of the Faculty of Education and Rehabilitation Sciences (University of Zagreb) approved the study and a written support from Croatian Ministry of Education, Science, and Sport and Croatian Teacher Training Agency was received. According to the Croatian Code of Ethics for Research with Children (Ajdukovic and Kolesaric, 2003) all participants who are older than 14 (as in this study) can give their own personal written or oral consent to participate in a study (Article 3, paragraph 3.4). In accordance with the Code we informed all participants about the main aim of the study and their voluntary participation in it. Consent to participate was considered implicit by the return of the questionnaire, after the participants were fully informed about the study. Additionally, all participants gave their oral informed consent to participate. Students could decline participation at any point during instrument administration.

## Author contributions

AH, DH, NR, and VK contributed to the study design, questionnaire development, administering, correcting, and entering questionnaires in the database. AH and DH were responsible for writing the introductory sections of the manuscript and prepared the article and all additional materials for submission. AH was responsible for statistical analysis. All authors contributed to the interpretation and discussion of the results. AH, DH, NR, and VK were responsible for the revision of the article and addressing the reviewer's comments and suggestions. All authors critically revised the work and approved the final version.

## Funding

The presented study is part of the wider project “Youth gambling in Croatia” which is financially and organizationally supported by the Ministry of Science, Education and Sports of the Republic of Croatia, Education and Teacher Training Agency, and Croatian Lottery, Ltd.

### Conflict of interest statement

The authors declare that the research was conducted in the absence of any commercial or financial relationships that could be construed as a potential conflict of interest.
